# Differentially activated B cells develop regulatory phenotype and show varying immunosuppressive features: a comparative study

**DOI:** 10.3389/fimmu.2023.1178445

**Published:** 2023-09-05

**Authors:** Elina A. Zheremyan, Alina S. Ustiugova, Aksinya N. Uvarova, Nina M. Karamushka, Ekaterina M. Stasevich, Violetta S. Gogoleva, Apollinariya V. Bogolyubova, Nikita A. Mitkin, Dmitry V. Kuprash, Kirill V. Korneev

**Affiliations:** ^1^ Center for Precision Genome Editing and Genetic Technologies for Biomedicine, Engelhardt Institute of Molecular Biology, Russian Academy of Sciences, Moscow, Russia; ^2^ Faculty of Biology, Lomonosov Moscow State University, Moscow, Russia; ^3^ Laboratory of Transplantation Immunology, National Medical Research Center for Hematology, Moscow, Russia

**Keywords:** regulatory B cells, Breg induction *ex vivo*, mBregs, tBregs, primary B cell activation, immunosuppression, immune response regulation, adoptive Breg transfer

## Abstract

Regulatory B lymphocytes (Bregs) are B cells with well-pronounced immunosuppressive properties, allowing them to suppress the activity of effector cells. A broad repertoire of immunosuppressive mechanisms makes Bregs an attractive tool for adoptive cell therapy for diseases associated with excessive activation of immune reactions. Such therapy implies Breg extraction from the patient’s peripheral blood, *ex vivo* activation and expansion, and further infusion into the patient. At the same time, the utility of Bregs for therapeutic approaches is limited by their small numbers and extremely low survival rate, which is typical for all primary B cell cultures. Therefore, extracting CD19^+^ cells from the patient’s peripheral blood and specifically activating them *ex vivo* to make B cells acquire a suppressive phenotype seems to be far more productive. It will allow a much larger number of B cells to be obtained initially, which may significantly increase the likelihood of successful immunosuppression after adoptive Breg transfer. This comparative study focuses on finding ways to efficiently manipulate B cells *in vitro* to differentiate them into Bregs. We used CD40L, CpG, IL4, IL21, PMA, and ionomycin in various combinations to generate immunosuppressive phenotype in B cells and performed functional assays to test their regulatory capacity. This work shows that treatment of primary B cells using CD40L + CpG + IL21 mix was most effective in terms of induction of functionally active regulatory B lymphocytes with high immunosuppressive capacity *ex vivo*.

## 1 Introduction

Regulatory B cells (Bregs), major regulators of immune system homeostasis, are known to be directly involved in the pathogenesis of rheumatoid arthritis, psoriasis, multiple sclerosis, type 1 diabetes, Sjögren’s syndrome, systemic lupus erythematosus, myocarditis, allergies, bacterial and viral infections, cancers, and graft versus host reaction (1–4). Reduced Breg pool has been shown in patients with chronic inflammatory diseases, autoimmune pathologies, and asthma (5, 6). Oliveria and colleagues demonstrated that regulatory B cells display distinct dysregulation in healthy volunteers compared to both allergic patients with and without asthma (7, 8). Elevated Bregs have been observed in cancer and acute bacterial infections (9, 10). Recently, COVID-19 patients have been shown to have increased levels of Bregs in their blood compared to healthy donors (11). In addition, the ability of certain subpopulations of Bregs to suppress effector T lymphocytes and NK cells in the tumor microenvironment has been convincingly demonstrated to facilitate tumor immune escape (9, 12).

Subpopulations of regulatory B cells can be subdivided into more than ten subgroups (13). However, when investigating the role of regulatory B cells in various pathologies, the most common subpopulations of human Bregs to be studied are memory Bregs (mBregs, CD19^+^CD24^hi^CD27^+^) and transitional Bregs (tBregs, CD19^+^CD24^hi^CD38^hi^), because of their high immunosuppressive capacity (14). One of the hypothetical models of Breg development is based on the assumption that any B cell can differentiate into Breg depending on specific stimuli (15), as suggested by experimental induction of Breg differentiation using various stimulation agents (16, 17). Differentiation of Bregs requires BCR signaling and the CD40-CD40L interaction (18). CD40-CD40L interaction occurs between B and T cells, respectively, and normally induces the formation of antibody-secreting cells. However, excessive duration of this signal inhibits the differentiation to plasma cells and leads mainly to the generation of Bregs. The role of CD40 in the induction of Bregs was confirmed in experiments on the mice model with CD40-deficient B lymphocytes. These mice exhibited a severe form of experimental autoimmune encephalomyelitis, accompanied by decreased IL10 production and increased Th1 and Th17 responses (19, 20).

Toll-like receptor (TLR) signaling is also important for Breg activation and, together with CD40, for their differentiation since TLR ligands are known to be strong promoters of Breg development (21). TLRs play a key role in the body’s defense against pathogens and may have protective value in autoimmune pathologies. Agonist stimulation through TLR4 (using LPS) and/or TLR9 (using CpG) has been shown to reduce symptoms of diabetes, EAE, and arthritis in mice, while decreased TLR9 expression in humans leads to an increased incidence of systemic lupus erythematosus (22–25). Bregs possess a unique spectrum of immunosuppressive mechanisms, which includes soluble factors (IL10, IL27, IL35, TGFβ, granzyme B) and surface molecules (PD-L1, FasL, CD39, CD73) (26–32). Such a broad repertoire of immunosuppressive mechanisms makes the adoptive transfer of Bregs a promising therapeutic approach for diseases associated with excessive activation of immune responses (33–35). At the same time, small numbers of Bregs and their extremely low survival rate in culture complicate the development of therapeutic applications.

There are various approaches for increasing the survival of B cells *in vitro*. For a long time, activation and proliferation of B cells *in vitro* were achieved by feeder cell culture systems expressing CD40L on the plasma membrane (36). CD40L binding to CD40 expressed on B cells mimics the signal from follicular T helper cells. By expressing CD40L and IL21, these cells give B lymphocytes a strong costimulatory signal. Many studies have shown feeder systems to be effective, but the standardization of assay protocols is quite complicated. Consequently, the introduction of soluble CD40L into cell culture has begun (37). CD40L can be replaced with an agonistic antibody to cross-link CD40 on the cell surface. CD40L is often used together with antibodies to the B cell receptor (BCR) in order to mimic BCR binding to an antigen (38). Activation using CD40L together with anti-BCR antibodies mostly promotes the proliferation of naive B cells (38), whereas the addition of IL21 to the activation medium promotes the proliferation of all B cell subtypes (39). It should be noted that CD40L + IL21 stimulation could promote B lymphocyte differentiation into germinal center B cells (40) which are characterized by rapid division and death *ex vivo*, as well as the potential ability to give rise to new CD27^+^ memory B cells.

IL4 and IL21 have also found application in *in vitro* B cell culture (41). It has been shown that the addition of IL4 promotes better survival of non-switched memory cells (42), while IL21, in contrast, promotes differentiation into a more mature state, which is accompanied by an increase in the number of plasma cells and switched memory cells (43). IL4 is considered important for B cell survival, while IL21 is more important for B cell differentiation (44). TLR antagonists can also be used to activate B cells *in vitro*. Stimulation of TLR9 with CpG was shown to promote the proliferation and differentiation of memory B cells, whereby cells with switched antibody isotype were more activated than non-switched ones (38).

In order to exhibit suppressive functions, B cells require inflammatory stimuli (via TLRs), costimulatory signals (via CD40), and cytokines (IL4, IL21, etc.) (45). Several approaches enhance the survival of Bregs under *in vitro* conditions. Glass et al. conducted a comprehensive phenotypic analysis of IL10^+^ B cells induced by a variety of exogenous stimuli in *ex vivo* cell culture experiments (46). The most popular stimulating agents for Breg culture are CpG, CD40L, and phorbol-12-myristate-13-acetate (PMA) in various combinations. Chen and colleagues, in their work on Bregs in patients with thyroid-associated ophthalmopathy, used combinations of CD40L and CpG to enrich peripheral blood mononuclear cells (PBMCs) with CD19^+^IL10^+^ Breg fraction (47). Iwata and colleagues studied the induction of PBMC differentiation into B10 cells (IL10-expressing B cells) by the following stimulating agents: lipopolysaccharide, CD40L, antibody to CD40, CpG, PMA, and ionomycin in various combinations (48). In another study, B lymphocytes isolated from PBMCs were activated with CpG and demonstrated an increased suppression of T lymphocyte activity due to secretion of the anti-inflammatory cytokine TGFβ and indoleamine-2,3-dioxygenase (27). Bankó et al. showed that B cells isolated from rheumatoid arthritis patients can be stimulated by CpG + CD40L for the induction of IL10 response. This effect can be boosted by adding IL21 to culture medium (49). This work aims to assess the applicability of such approaches to the preparation of biomedical cell products for adoptive Breg therapy.

## 2 Materials and methods

### 2.1 Human subjects

Current study was performed on healthy human subjects who donated their blood at the National Medical Research Center for Hematology (Moscow, Russia). The study was approved by the Research Ethics Committee of the National Medical Research Center for Hematology (Protocol № 126, 25.02.2022). All donors signed the informed consent form before enrollment.

### 2.2 Isolation of B cells from PBMCs

PBMCs were isolated by Ficoll-Hypaque (1.077 g/cm³, Paneco, Russia) density gradient centrifugation (400g, 30 min). B cells were purified by magnetic separation using the CD19-MicroBeads (Miltenyi Biotec, Germany). Cells were cultured in full RPMI-1640 medium supplemented with 2mМ glutamine (Paneco), 20% FBS (Biosera, France), 1 mМ sodium pyruvate, 10 mМ HEPES, 100 U/ml penicillin, and 100 mcg/ml streptomycin (all Paneco).

### 2.3 Activation of B cells

B cells were cultured in the activation medium for 7 days. Among activation agents, there were recombinant CD40L (1 mcg/ml, BioLegend, USA), IL4 (10 ng/ml), IL21 (25 ng/ml, all Miltenyi Biotec), PMA (10 ng/ml) and ionomycin (1 mcg/ml, all Sigma, USA) and CpG (2 mcM, ODN2006, 5’-tcgtcgttttgtcgttttgtcgtt-3’), in various combinations. Negative control was maintained in the growth medium only. On day 7, supernatants were collected separately.

### 2.4 B cell immunophenotyping

Cells were stained with antibodies to the main phenotypic molecular markers of regulatory B cells: CD24-PE (clone ML5, lot No. B273849), CD27-PE-Cy7 (clone O323, lot No. B274956), CD38-APC (clone HIT2, lot No. B255162) (all BioLegend). To determine cell viability, cells were also stained with Viability Dye-eFluor780 (Thermo Fisher Scientific, USA). Cells were then analyzed by flow cytometry using FACS Canto II (BD Biosciences, USA). FlowJo Software version 10 (TreeStar, USA) was used for analysis.

### 2.5 Breg proliferation assay

Magnetically separated B cells were labeled with CellTrace Violet (CTV, cat No. C34557, Thermo Fisher Scientific) and cultured (10^5^ cells) in the activation medium for 7 days. Negative control was maintained in full RPMI-1640 without any additional stimuli. After 7 days of cultivation, cells were then phenotyped as described above.

### 2.6 Analysis of anti-inflammatory gene expression level

Total RNA was purified from activated B cells on day 7 with the ExtractRNA reagent (Evrogen, Russia). cDNAs were synthesized using oligo(dT)_18_ primers with MMLV RT kit (Evrogen), and mRNA expression was determined with the Bio-Rad Real-time CFX96 Touch (Bio-Rad, USA) using a qPCRmix-HS SYBR kit (Evrogen). The ΔΔСt method (50) was used to normalize transcription to beta-actin (*ACTB*). The following primer pairs were used: *CD274* - forward 5’-TGCAGGGCATTCCAGAAAGA-3’ and reverse 5’-TAGGTCCTTGGGAACCGTGA-3’, *EBI3* - forward 5’-GCTCCCTACGTGCTCAATGT-3’ and reverse 5’-CCCTGACGCTTGTAACGGAT-3’, *ACTB* - forward 5’-ACTGGGACGACATGGAGAAA-3’ and reverse 5’-GGCGTACAGGGATAGCACAG-3’.

### 2.7 Determination of pro- and anti-inflammatory cytokine levels secreted by B cells

Supernatants from activated B cells were collected separately on day 7. Concentrations of pro- and anti-inflammatory cytokines TNF and IL10 were determined by ELISA (cat No. 88-7346-88 and 88-7106-88, Thermo Fisher Scientific).

### 2.8 Effector cell proliferation assay

Before the experiment, CD19-depleted PBMCs were maintained in IL2-containing (20 ng/ml, SCI-Store, Russia) full RPMI-1640 medium. Then autologous CTV-labeled (cat No. C34557, Thermo Fisher Scientific) CD19-depleted PBMCs (10^5^ cells) were co-cultured with previously differentially activated and non-activated B cells (10^5^ cells). After 5 days of co-incubation, supernatants were collected separately to determine pro- and anti-inflammatory cytokine levels by ELISA (cat No. A-8752 for IFNγ, A-8756 for TNF, A-8766 for IL1β, A-8768 for IL6, and A-8774 for IL10, Vector-Best, Russia). Cells were stained with antibodies to the main phenotypic markers of NK- and T cells: CD3-FITC (Sorbent, Russia), CD4-APC (clone RPA-T4, lot No. B307926, BioLegend), CD8-PE-Cyanine7 (clone SK1, lot No. B276851, BioLegend), CD16-PE (Sorbent), cell viability was determined using Viability Dye-eFluor780 (Thermo Fisher Scientific). Frequencies of proliferating CD3^-^CD16^+^ NK-cells, CD3^+^CD4^+^ T helpers, CD3^+^CD8^+^ T killers were determined by flow cytometry following the loss of CTV signal to determine the suppressive capacity of B cells. Cells were then analyzed by flow cytometry using a FACS Canto II (BD Biosciences, USA), FlowJo software version 10 (TreeStar) was used for analysis.

### 2.9 Measurement of NK-mediated cytotoxic activity by monitoring cancer cell adhesion and apoptosis using the xCELLigence RTCA system

MCF7 and NKL cell lines were used for this experiment. MCF7 adhesive human breast cancer cells (provided by late Dr. E. Zabarovsky from Karolinska Institutet, Stockholm, Sweden) were cultured in full DMEM (Paneco). NKL natural killer cells (provided by Dr. M. Streltsova and Dr. A. Sapozhnikov from the Institute of Bioorganic Chemistry, Moscow, Russia) were maintained in full RPMI-1640 supplemented with IL2 (20 ng/ml, SCI-Store). MCF7 cells were seeded in each well of the E-plate (5*10^4^ cells per well). The impedance values of individual cells were automatically monitored by the xCELLigence system (xCelligence DP, Agilent, USA) and expressed as a cell index (CI) value (51). Meantime, NKL cells were co-cultured with differentially activated and non-activated B cells overnight. When MCF7 reached the steady-state growth phase, NKL (2*10^5^ cells) and B cells (2*10^5^ cells) were seeded onto the E-plate at a density of 10^5^ per well. The E-plate was then placed into the xCELLigence system. Sweeps were taken every 15 minutes for the duration of the experiment to determine the MCF7 adhesion level, which was indicative of NK-dependent cancer cell cytolysis. The cell index was normalized to 1.0 at the moment of NKL addition.

### 2.10 B-cell-mediated Treg-differentiation

CD4^+^ T cells were magnetically separated from PBMCs (CD4-MicroBeads, Miltenyi) and maintained in IL2-containing (20 ng/ml, SCI-Store) full RPMI-1640 medium until the start of the experiment. Then they were labeled with CTV (cat No. C34557, Thermo Fisher Scientific) and co-cultured (10^5^ cells) with autologous previously differentially activated for 5 days and non-activated B cells (10^5^ cells). After 5 days of co-incubation, supernatants were collected separately for determination of IL10 level by ELISA (cat No. 88-7106-88, Thermo Fisher Scientific). Cells were stained with antibodies to the main phenotypic molecular markers of regulatory T cells: CD4-APC (clone RPA-T4, lot No. B307926, BioLegend), CD25-PE (clone BC96, lot No. 2173867, eBioscience), and CD127-FITC (clone A019D5, lot No. E-AB-F1152L, Elabscience, USA). To determine cell viability, cells were also stained with Viability Dye-eFluor780 (Thermo Fisher Scientific). Cells were then analyzed by flow cytometry using FACS Canto II (BD Biosciences), FlowJo Software version 10 (TreeStar) was used for analysis.

### 2.11 Statistical analysis

All analysis was performed by GraphPad Prism, version 9.0.0. (GraphPad Software Inc., La Jolla, CA, USA). Results are expressed as mean ± SEM.

## 3 Results

### 3.1 Breg ratio, viability, and proliferation rates differ under the influence of various stimuli

**Figure 1:**
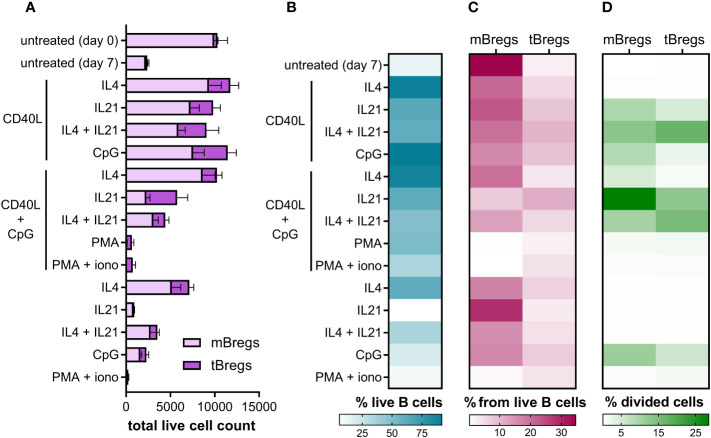
Differential activation of primary CD19^+^ lymphocytes alter the Breg subsets ratio, their viability, and proliferation rates *ex vivo*. **(A)** Total live cell count of mBregs and tBregs after various stimulation (50,000 events were acquired). Untreated control values were measured on day 0 and day 7. Mean values ± SEM of five independent experiments for each condition are shown. **(B)** Heatmap showing average B cell viability percentages after various stimulation. The mean values of five independent experiments for each stimulation are shown. **(C)** The ratio of mBregs and tBregs among live B cells after activation with various stimuli for 7 days. Untreated control values were measured on day 0 and day 7. Mean values of five independent experiments for each condition are shown. **(D)** Heatmap showing the average percentage of divided mBregs and tBregs induced by differential activation of primary B cells. The mean values of five independent experiments for each stimulation are shown.

Since CD24^hi^CD38^hi^ and CD24^hi^CD27^+^ B lymphocytes are known to be the major Breg subsets in human peripheral blood (14), we used them as an analytical instrument to assess Breg percentage among differentially activated CD19^+^ lymphocytes (B cells) using flow cytometry analysis (**Figure S1**, **Supplementary Data**). mBregs show a higher survival rate compared to the tBreg subpopulation (**Figures 1A, C**). This feature may be explained by their memory phenotype, which makes them more persistent and ensures their longevity (52). Transitional B cells require more environmental factors to survive (53), thus showing lower survival rates without stimulation *in vitro* (**Figure 1**).

We observed that the majority of activation agents (such as the combination of CD40L + IL4, CD40L + CpG, etc.) lead to a higher frequency of mBregs compared to tBregs (**Figures 1A, C**). At the same time, PMA or PMA + ionomycin, alone, or in combination with CD40L + CpG, abrogated the acquisition of a regulatory phenotype by peripheral blood B cells, which is especially pronounced for the mBreg subpopulation. Activation of B cells by CD40L, CpG, and IL4 in various combinations leads to increased survival, but supplementing the activation medium with IL21 partially lowers cell viability (**Figure 1B**).

In order to assess the rates of Breg proliferation under various stimuli, we used CellTrace Violet staining to monitor distinct generations of proliferating cells by dye dilution (**Figure S1**). This assay has revealed that under various stimuli mBregs and tBregs proliferate at different rates (**Figure 1D**). While most stimuli induced the predominant proliferation of mBregs, supplementation of the CD40L-containing activation mix with IL21 increased the growth of both mBregs and tBregs. CpG also positively influenced their proliferation rate but to a smaller extent. Some activation agents, such as PMA, ionomycin, or interleukins alone, did not induce the proliferation of Breg subpopulations. The results also revealed that IL4 in combination with CD40L + CpG, or CD40L alone, enhanced primary B cell survival, but hardly induced the proliferation of either mBregs or tBregs.

### 3.2 Expression of major molecules involved in Breg-mediated immunoregulation

**Figure 2:**
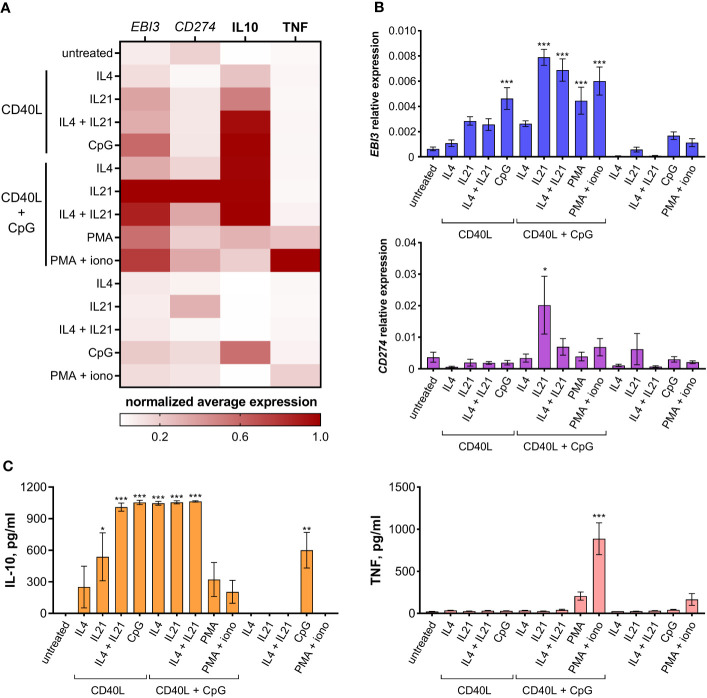
Immunoregulatory molecular profile of differentially activated B cells. **(A)** Heatmap showing *EBI3*, *CD274*, IL10, and TNF normalized average expression levels in differentially activated B cells. **(B)** *EBI3* and *CD274* relative expression levels were determined by RT-PCR using total RNA extracted from differentially activated B cells on day 7 of cultivation. **(C)** IL10 and TNF levels were determined by ELISA in the medium from differentially activated B cells on day 7 of cultivation. Mean values ± SEM of at least four independent experiments is shown. *P < 0.05, **P < 0.01, ***P < 0.001 compared to untreated control, as calculated by ANOVA.

We next analyzed the immunoregulatory molecular profile of differentially activated B cells. Regulatory B cells are known to express a variety of inhibitory molecules – both membrane-bound factors, such as PD-L1, and soluble molecules, such as IL10, IL35, etc. We tested differentially activated B cells for IL10 secretion levels and the expression of anti-inflammatory genes *CD274* (coding for PD-L1) and *EBI3* (coding for IL27B – a common subunit of IL35 and IL27 cytokines); TNF production by B cells was assessed to monitor inflammatory responses (**Figure 2**). All three inhibitory molecules tested were expressed at the highest level by B cells treated with CD40L + CpG + IL21, making this combination of stimuli the most efficient in inducing an anti-inflammatory response. IL10 was expressed at the same high level in B cells treated with CD40L in several combinations with CpG, IL21, and/or IL4. The usage of PMA and ionomycin as stimulation agents increased the level of *EBI3*, *CD274*, and IL10 expression. However, this treatment also resulted in an undesirable increase in TNF level, especially in combination with CD40L and CpG.

### 3.3 Activation-induced regulatory B cells mediate Treg differentiation

Based on expression levels of inhibitory molecules (**Figure 2**) and rates of Breg proliferation (**Figure 1**), we have selected 5 combinations of activation stimuli for further functional analysis: CD40L + CpG, CD40L + CpG + IL4, CD40L + CpG + IL21, CD40L + CpG + IL4 + IL21, and CpG alone.

**Figure 3:**
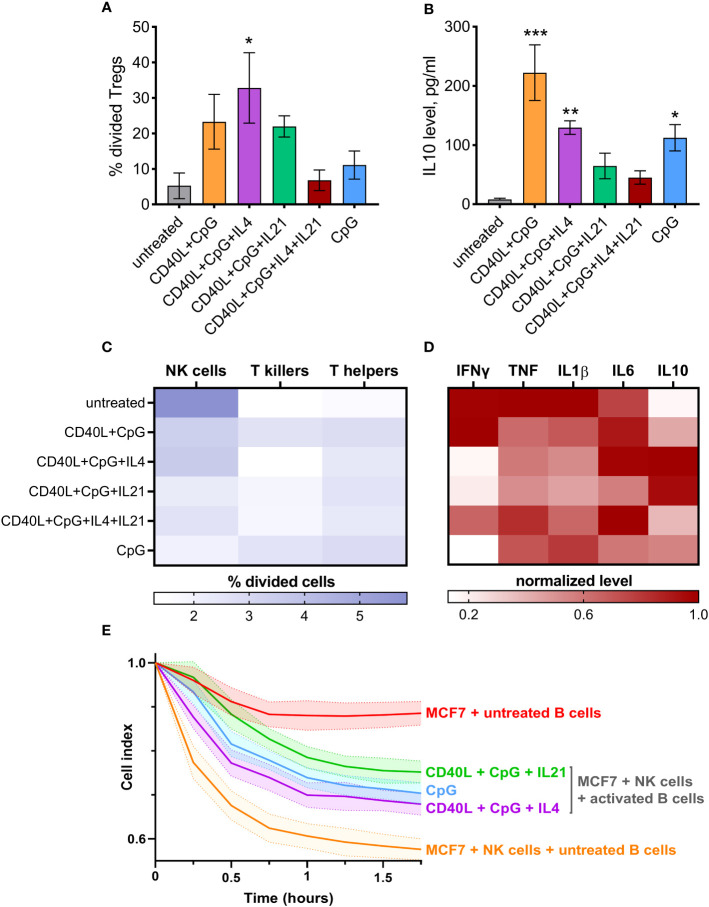
Differentially activated B cells suppress the activity of effector cells and mediate CD4^+^ T cells differentiation into Tregs (CD4^+^CD25^hi^) with simultaneous induction of IL10 secretion. **(A)** Tregs proliferation induced by co-cultivation of differentially activated B cells and CD4^+^ T cells. The percentage of divided Tregs was determined by flow cytometry using CellTrace Violet dye. **(B)** IL10 level was determined in the medium from CD4^+^ T cells co-cultivated with differentially activated B cells on day 5 of cultivation using ELISA. Mean values ± SEM of four independent experiments is shown. *P < 0.05, **P < 0.01, ***P < 0.001 compared to untreated control, as calculated by ANOVA. **(C)** Activated B cells influence the proliferation rate of T helpers, T killers, and NK cells from CD19-depleted PBMCs after 5 days of co-cultivation. **(D)** IFNγ, TNF, IL1β, IL6, IL10 normalized levels were determined using ELISA in the medium from B cell-depleted PBMCs after co-cultivation. The average values of four independent experiments are shown. **(E)** Activated B cells reduce NK-mediated killing of cancer cell line MCF7. Mean values ± SD of three independent experiments is shown.

Regulatory immune cells are known to significantly influence nearby immune cells, making them acquire a suppressive phenotype. To assess the capacity of activated B cells to induce regulatory T cell (Treg) differentiation, we used a co-cultivation assay followed by an evaluation of Treg proliferation rate using CTV staining (**Figure S1**). These Tregs are likely to be peripherally induced Tregs (pTregs) since pTregs are inducible within suppressive microenvironments, which are precisely created by Bregs (54). Our data revealed that B cells stimulated with CD40L + CpG + IL4 efficiently induced the proliferation of Tregs (gated as CD4^+^CD25^hi^ T cells) (**Figure 3A**). B cells treated with CD40L + CpG and CD40L + CpG + IL21 also mediated Treg proliferation, but to a lesser extent. CD4^+^CD25^hi^ T cells were further verified for low expression of CD127 (**Figure S2**), which is characteristic of Tregs (55). IL10 levels in the culture medium demonstrated a similar pattern, however, the use of B cells treated with CD40L + CpG resulted in the highest level of this anti-inflammatory cytokine (**Figure 3B**). Thus, activation-induced Bregs induce the immunosuppressive response in T helper cells, as demonstrated by the increase in Treg proliferation and IL10 secretion.

### 3.4 Activation-induced Bregs modify effector cell functioning

We further tested the ability of *in vitro*-stimulated B cells to modulate proliferation rates of NK cells, CD4^+^ T cells (T helpers), and CD8^+^ T cells (T killers) (**Figure S1**). B cells activated using CD40L + CpG + IL21, or CpG alone, had the highest inhibitory effect on the growth of NK cells (gated as CD3^–^CD16^+^). T helper and T killer proliferation rates were not suppressed by differentially activated B lymphocytes; some B cells (such as CD40L + CpG treated cells) even promoted their proliferation (**Figure 3C**). With regard to the major subpopulations of T cells, a slight decrease in the percentage of T killers can be observed under the influence of activated B cells (**Figure S3**). This phenomenon may be attributed to the regulatory ability of B cells to suppress T killers (56). Furthermore, a minor increase in the percentage of T helpers is detected, which could be associated with the proliferation of Tregs induced by suppressive B cells (**Figures 3**, **S2, S3**). We also assessed the level of the common effector cytokine IFNγ, pro-inflammatory TNF, IL1β, IL6, and anti-inflammatory IL10 secreted by CD19-depleted PBMCs cultured with treated B cells for 5 days. B cells treated with CD40L + CpG + IL21/IL4, or with CpG alone, decreased inflammatory response, as assessed by IFNγ levels (**Figure 3D**). B cells stimulated with CD40L + CpG + IL21/IL4 increased production of IL10 which also helped reduce the inflammatory reaction. Production of TNF and IL1β did not change dramatically, but the pattern of their expression resembled the IFNγ production. At the same time, the level of IL6 cytokine showed no clear pattern of changes in expression, presumably due to its bipolar proinflammatory and homeostatic nature (**Figure 3D**). In order to test activated B cells in a more physiological setting, we used the xCELLigence system for real-time monitoring of NK cell-mediated cytotoxicity against MCF7 breast cancer cells (**Figure 3E**). This analysis showed that the above-mentioned combinations of activation agents induced B cells to significantly inhibit the cytotoxic activity of NK cells. Thus, Bregs generated *in vitro* proved to possess immunosuppressive effects on NK cells and, probably, some other effector cells from human peripheral blood, which have not been examined in this study (based on the increased level of IL10 and decreased IFNγ, TNF, IL1β expression by B cell-depleted PBMCs, **Figure 3D**).

## 4 Discussion

**Figure 4:**
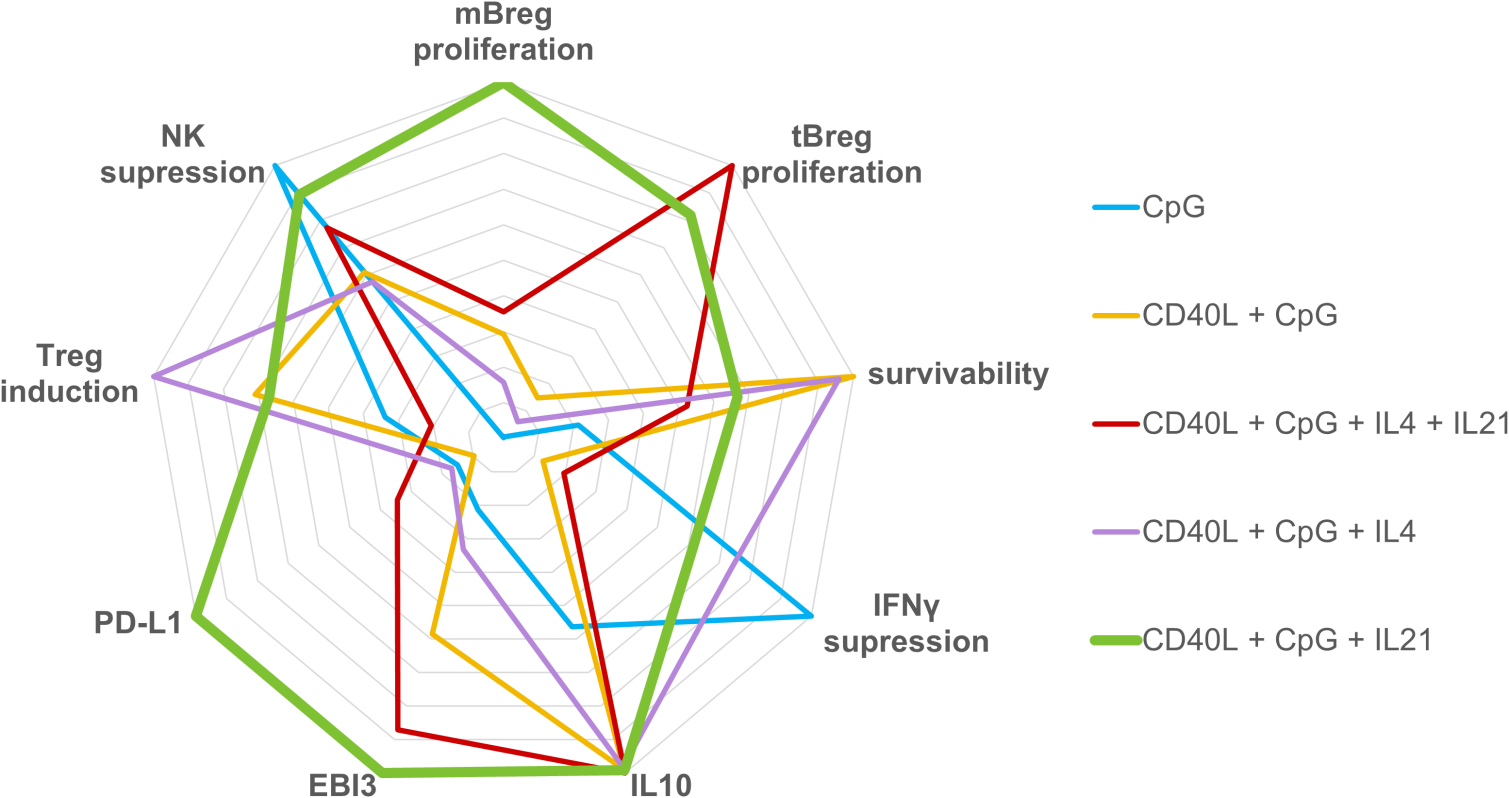
Radar graph representing the regulatory phenotype of B cells induced by various activation stimuli. Axes display immunosuppressive parameters of activated B lymphocytes assessed in the study. All data from **Figures 1**-**3** used for immunogram construction were proportionally normalized to a single scale from 0 to 10. The values of each axis have been joined to form the central polygon area, which represents the general regulatory phenotype induced by various activation cocktails.

Our analysis demonstrated that different combinations of activators have quite diverse effects on the separate components of the regulatory phenotype of B cells *in vitro*. As can be seen from **Figure 4**, which visualizes our findings, of the five “semi-finalists” selected based on proliferation and expression data, the CD40L + CpG + IL21 activation cocktail appeared to be the most universal mix overall. The selected activation mix was a bit less effective than other combinations in terms of cell survival (where CD40L + CpG mix apparently worked better without additional cytokines, **Figure 1B**), and Treg induction (where IL4, a cytokine with known immunosuppressive properties, had the edge over IL21, **Figure 3**). At the same time, after such activation, B cells didn’t show signs of the inflammatory response: the level of TNF expression was not raised (**Figure 2C**). Activated B cells have also decreased the expression levels of IFNγ, TNF, IL1β and increased IL10 production by other peripheral blood cells and suppressed the proliferation of NK cells and their cytotoxic activity (**Figure 3**). Our findings are consistent with studies on the role of IL21 in Breg maturation, where effector Bregs could not be generated in the absence of IL21 or its receptor (57), and T follicular helper cells suggested themselves as the most probable normal source of IL21 for the induction of Breg differentiation (58). Spolski and Leonard suggested using IL21 to expand B regulatory cells *in vitro* prior to adoptive transfer into patients with multiple sclerosis to inhibit inflammatory response (59). Chesneau and colleagues used a mixture of CD40L + CpG + IL21 with added anti-BCR and IL2 to induce *ex vivo* expansion of granzyme B-expressing B cells with potent regulatory properties (60). We propose to use IL21 in combination with CD40L, a major activator of B cells, and CpG as a polyclonal innate stimulus, in order to generate Bregs with proven high broad-spectrum immunosuppressive capacities and proliferation rate (**Figure 4**), which could potentially allow to efficiently reduce the inflammatory response in the treatment of pathologic conditions associated with immune system overactivation using adoptive Breg therapy.

Adding IL21 to the cocktail partially lowers cell survival which is in agreement with the published data that IL21 can trigger activation-induced cell death via downregulation of anti-apoptotic Bcl-2 and Bcl-xL production, and upregulation of Cas-8 and Cas-3 expression (61–63). This may become an obstacle when preparing Bregs as a biomedical cell product for adoptive transfer. Nevertheless, under *in vivo* conditions, some survivability is probably worth sacrificing for the sake of optimal immunosuppressive capacity as long as this problem can be solved by enrichment of the fraction of live Bregs *via* cell sorting before the cell transfer. Stimulation of B cells with CD40L + CpG + IL21 resulted in an increase in the population of plasmablasts (**Figure S4**), which can be attributed to the potential saturation of the Breg compartment, given the regulatory function demonstrated for CD27^hi^CD38^hi^ cells (64). Following activation, the number of conventional memory B cells exhibited a notable reduction, suggesting their potential differentiation into plasmablasts. Furthermore, we observed proliferation of naive B cells upon exposure to activating stimuli, in agreement with the findings reported in the study conducted by Glass et al. (46).

It is important to clarify that direct administration of CpG, CD40L and IL21 *in vivo* is unacceptable due to the risk of systemic immune hyperactivation and inflammatory response. Instead, we suggest utilizing these agents solely *ex vivo* to target B cells, and subsequently introducing the resulting purified cell product into the patient. This approach allows for targeted intervention without excessive systemic immune disturbances.

**Figure 5:**
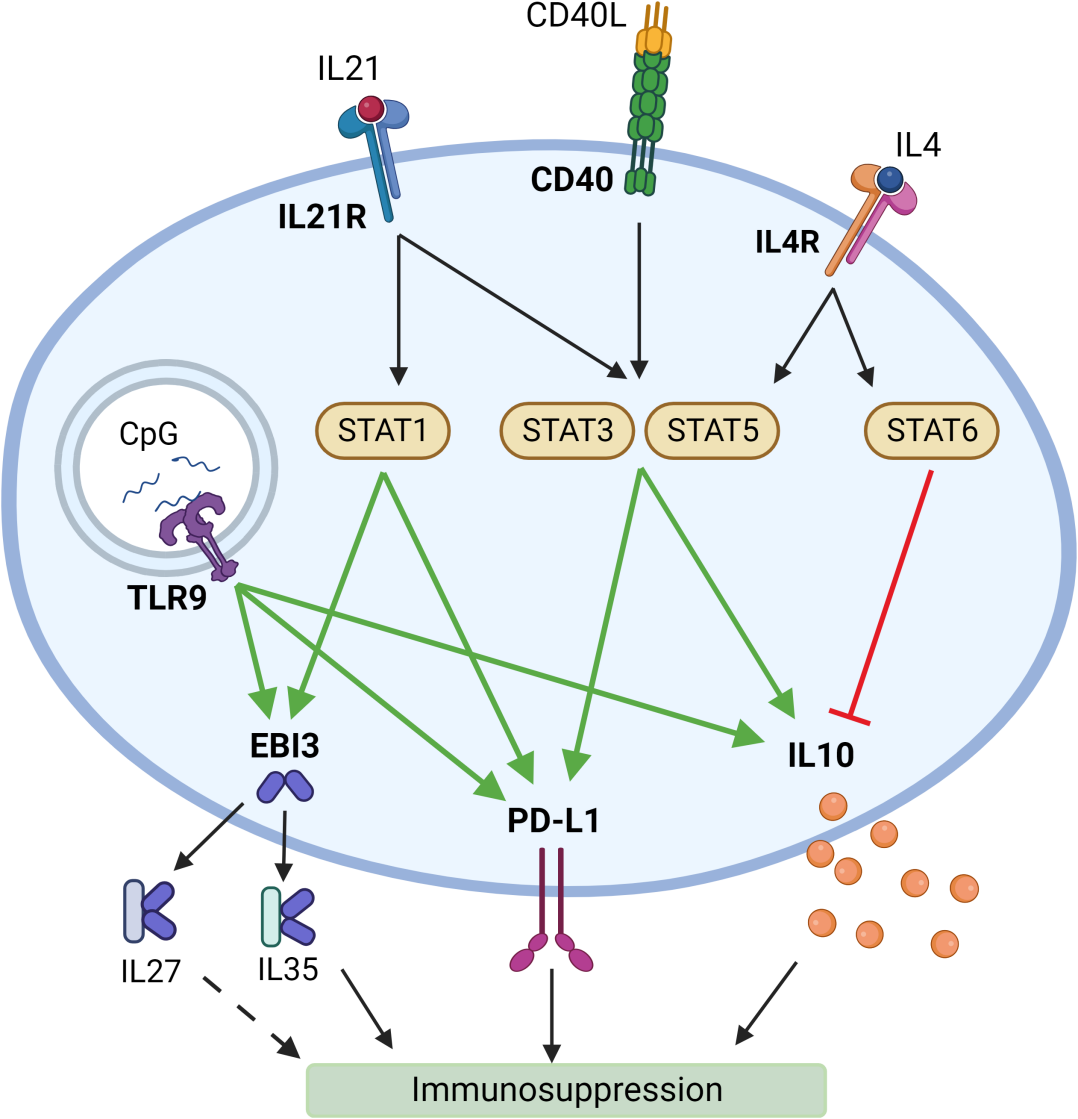
Potential mechanisms of induction of a suppressive phenotype in B lymphocytes by synergistic activation by different ligands. Created with BioRender.com.

Reflecting on the reasons why this combination of activation agents has proven to induce immunosuppressive response in B cells, we have noted that there is evidence that CD40L and IL21 signaling induces STAT3 (65, 66) and STAT5 (67, 68) activation, which in their turn may promote IL10 (65, 69) and PD-L1 expression (70). IL21 signaling was also found to activate STAT1 (71). Thus, it may be leading to the increase in EBI3 (72) and PD-L1 (73) expression. TLR9 – a receptor for CpG – has also been shown to promote IL10 (65), EBI3 (74), and PD-L1 (75) expression (**Figure 5**).

However, in pathologies associated with decreased Treg activity, a combination of CD40L, CpG, and IL4 may be more useful since Bregs activated this way significantly increased Treg proliferation (**Figure 3A**). It is known that the IL4R signaling pathway is involved in the differentiation of naive B cells in IL4-producing B cells (76). Thus, when *in vitro* stimulated with IL4, B cells can be induced to produce IL4 in an autocrine manner. It has also been shown that Treg differentiation depends on this cytokine: IL4 signaling can induce STAT6-dependent Foxp3 expression and maintenance of this transcription factor in Tregs (77). When co-cultivated with T helper cells, CD40L + CpG treated B cells induced IL10 secretion at a higher level compared to those treated with CD40L + CpG + IL4 mix (**Figure 3B**). Yet, there are many more mechanisms of Treg-mediated immunosuppression except for IL10 (78), which is why we suppose that such parameter as Treg proliferation rate is more indicative of the efficiency of Breg-inducing activation of B cells. Interestingly, during co-cultivation with CD19^+^-depleted PBMCs, B cells activated with CD40L + CpG + IL21/IL4 induced highest IL10 production (**Figure 3D**). This can be explained by the presence of other cells in PBMC that could have acquired an anti-inflammatory phenotype after such treatment – for example, monocytes (79).

CpG seemed to be an important player in the *in vitro* induction of Bregs; its addition to the stimulation mix has increased the secretion of inhibitory molecules (**Figure 2**) and contributed to a higher rate of Breg proliferation (**Figure 1D**). The work of Gallego-Valle and colleagues have also shown the effectiveness of CpG stimulation in the generation of Breg-like phenotype in B cells (80). However, CpG treatment alone has shown to be less effective than stimulation cocktails, consisting of CD40L, CpG, and IL4/IL21, primarily due to its low immunosuppression-inductive capacity (**Figure 3**).

Regarding PMA-containing cocktails, our results showed that such activation types weren’t successful in induction of immunosuppressive response. Interestingly, PMA alone or PMA + ionomycin-containing mixes are quite often used for the generation of Bregs *in vitro*, but in this study, they enhanced inflammatory response in B cells (based on the level of TNF secretion, **Figure 2**), didn’t induce either mBreg or tBreg proliferation, and even decreased survivability of primary B cells (**Figure 1**).

To gain insights into the distribution of Bregs in the body following adoptive cell transfer, we can consider the existing studies that have examined the *in vivo* assessment of infused Treg cells and their outcomes in humans (81). In a study involving four patients with autoimmune hepatitis, Tregs infused into blood were primarily found in the liver (22-44%), spleen (11-24%), and bone marrow (9-13%) (82). Bregs can also be expected to exhibit a predominant distribution towards the site of inflammation which would be consistent with the objective to target and suppress inflammation in a specific localized area rather than pursuing a systemic approach.

There are several limitations of our study that should be mentioned. One is our gating strategy for Bregs that is well-established but not the only one possible since a canonical regulatory Breg phenotype remains elusive in the absense of a specific Breg marker (14). Another limitation is inclusion of IL21 in the activation cocktail that appears to partially lower the cell survivability. However, in the context of potential adoptive therapy, this reduced survival may not be a significant issue and could even be advantageous, as it limits prolonged cell proliferation, thereby reducing the probability of non-specific immortalization and transformation into malignant B cells.

Within the above-mentioned limitations, our comparative study sheds light on efficient ways of generating functional Bregs from bulk peripheral blood B cells. Our study shows the high efficiency of the CD40L + CpG + IL21 activation mix in the induction of functionally active regulatory B cells *ex vivo*. Such activation could be performed on the peripheral blood B cells prior to adoptive cell transfer. We hope this research will bring new insights into the development of new approaches to the adoptive cell therapy of pathologies caused by immune overactivation, such as allergies, graft-versus-host disease, and autoimmune diseases.

## Data availability statement

The original contributions presented in the study are included in the article/**Supplementary Materials**, further inquiries can be directed to the corresponding authors.

## Ethics statement

The studies involving human participants were reviewed and approved by Research Ethics Committee of the National Medical Research Center for Hematology (Protocol № 126, 25.02.2022). The patients/participants provided their written informed consent to participate in this study.

## Author contributions

EZ, ASU, and KK designed research and analyzed data. EZ, ASU, ANU, NK, and KK performed experiments. ES, VG, AB, NM, and DK contributed to the critical expertise, materials, and techniques. EZ wrote the manuscript. DK and KK reviewed and edited the manuscript. All authors contributed to the article and approved the submitted version.
