# Supplementary material for: Differentially activated B cells develop regulatory phenotype and show varying immunosuppressive features: a comparative study
Source: Front Immunol. 2023 Sep 5;14:1178445. doi: 10.3389/fimmu.2023.1178445 (PMC10509016; doi:10.3389/fimmu.2023.1178445)
Supplement: Supplementary file 1 [file Presentation_1.pdf]

Supplementary Material

Differentially activated B cells develop regulatory phenotype and show varying immunosuppressive features: a comparative study

Elina A. Zheremyan[1,*], Alina S. Ustiugova[1], Aksinya N. Uvarova[1,2], Nina M. Karamushka[2], Ekaterina M. Stasevich[1], Violetta S. Gogoleva[1], Apollinariya V. Bogolyubova[3], Nikita A. Mitkin[1], Dmitry V. Kuprash[1,2], Kirill V. Korneev[1,3,*]

*** Correspondence:**
Elina A. Zheremyan: elyazheremyan@mail.ru
Kirill V. Korneev: kirkorneev@gmail.com

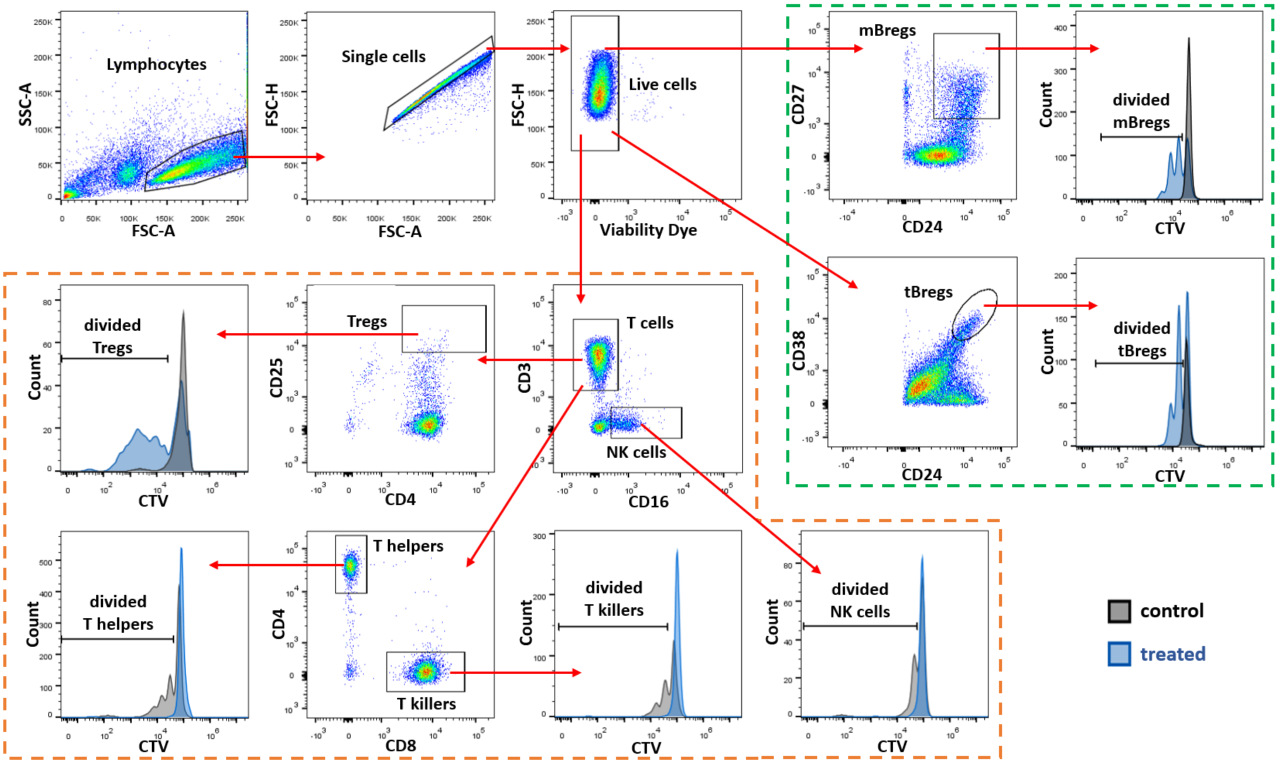

Figure S1. Flow cytometry gating strategies for the assessment of cell ratio and proliferation rates of Breg subsets and effector cells. **Right green area**: Identification of the ratio and cell division rates of mBreg ($CD24^{hi}CD27^{+}$) and tBreg ($CD24^{hi}CD38^{hi}$) subpopulations in live $CD19^{+}$ cells isolated by magnetic separation. **Left orange area**: Identification of the ratio and proliferation rates of effector lymphocytes: $CD3^{+}$ T cells ($CD8^{+}$ T killers, $CD4^{+}$ T helpers and $CD4^{+}CD25^{hi}$ regulatory T cells) and $CD3^{-}CD16^{+}$ NK cells in $CD19^{+}$-depleted PBMCs.

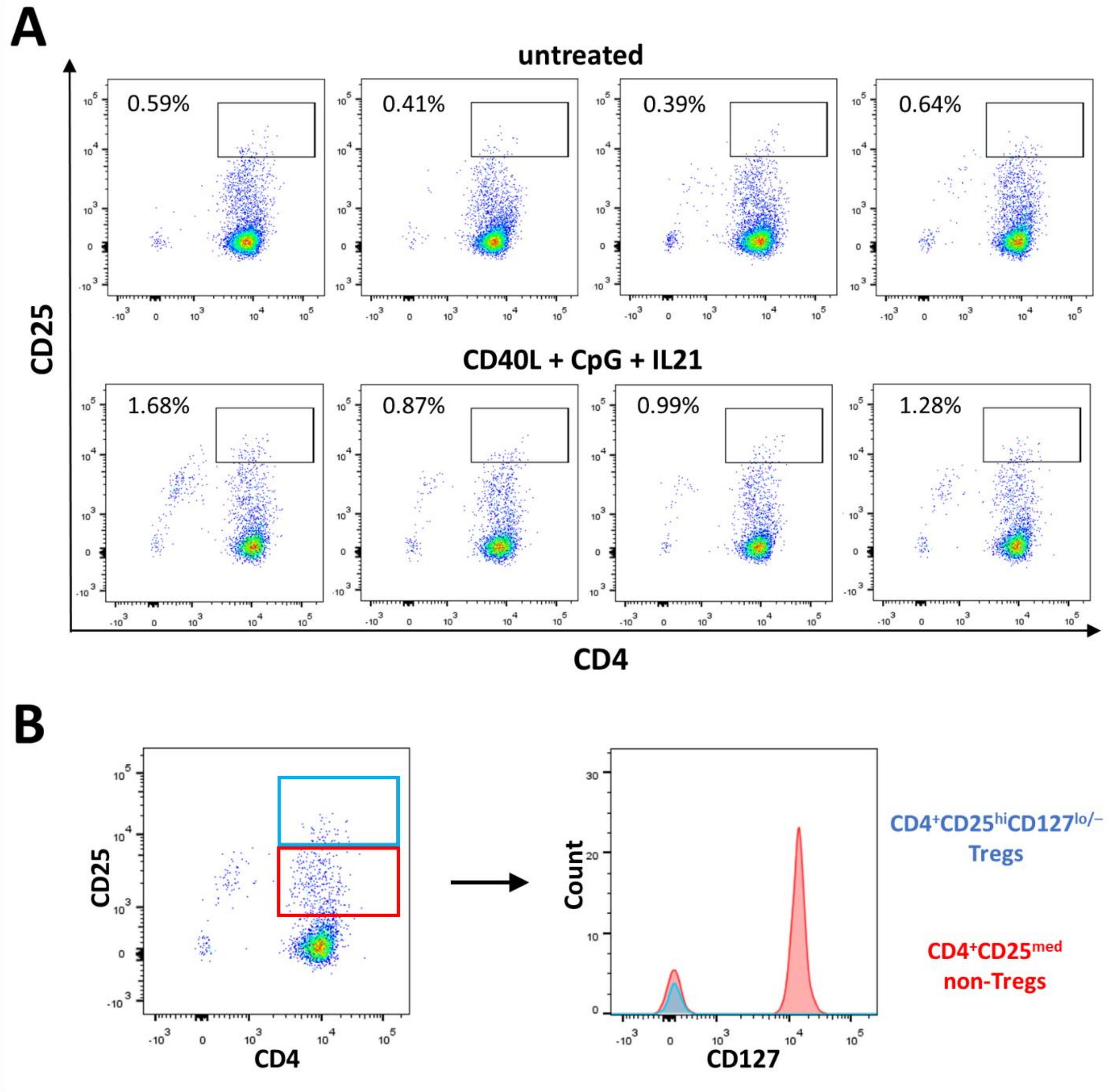

Figure S2. (A) The assessment of $CD4^+CD25^{hi}$ (regulatory T cells) ratio of $CD3^+$ T cells after 5 days of co-incubation of $CD19^+$-depleted PBMCs with activated (CD40L + CpG + IL21) and untreated B cells. (B) Verification of $CD4^+CD25^{hi}$ T cells for low expression of CD127, which is characteristic of Tregs.

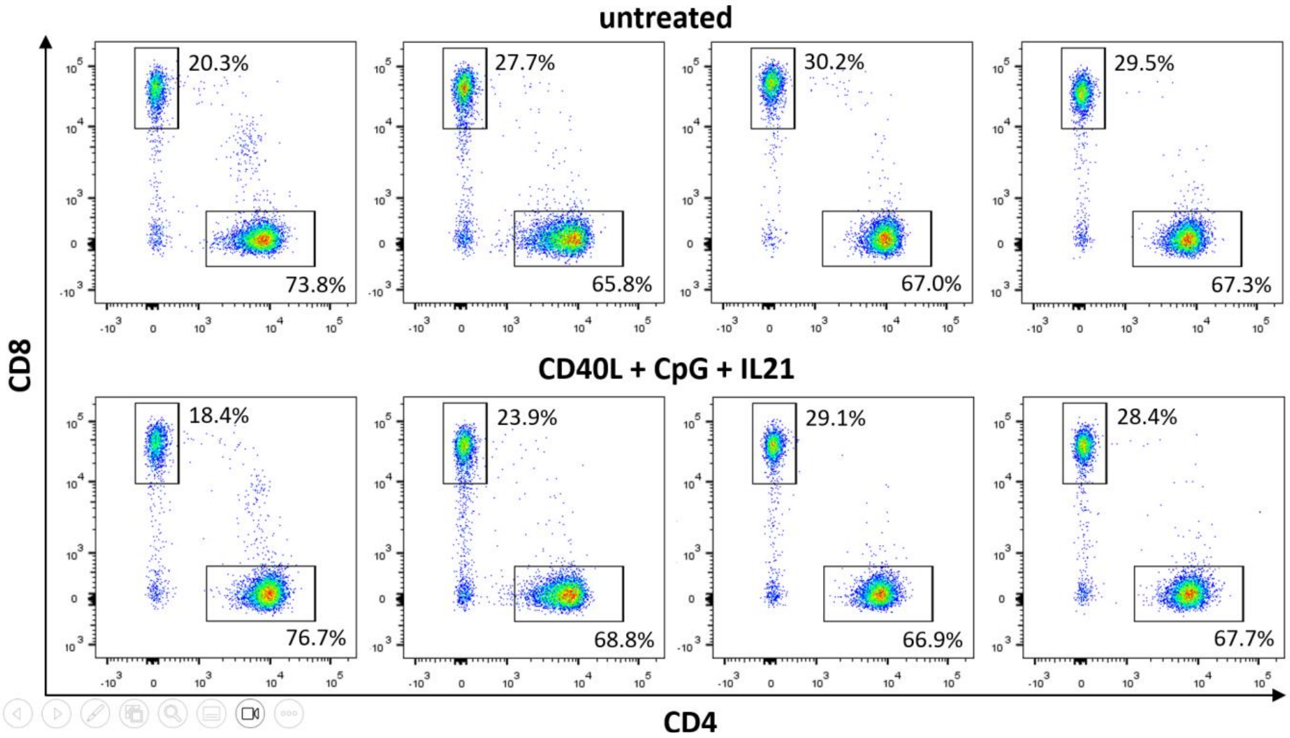

Figure S3. Distribution of $CD4^+$ (T helpers) and $CD8^+$ (T killers) T cell subsets after 5 days of co-incubation of $CD19^+$-depleted PBMCs with activated (CD40L + CpG + IL21) and untreated B cells.

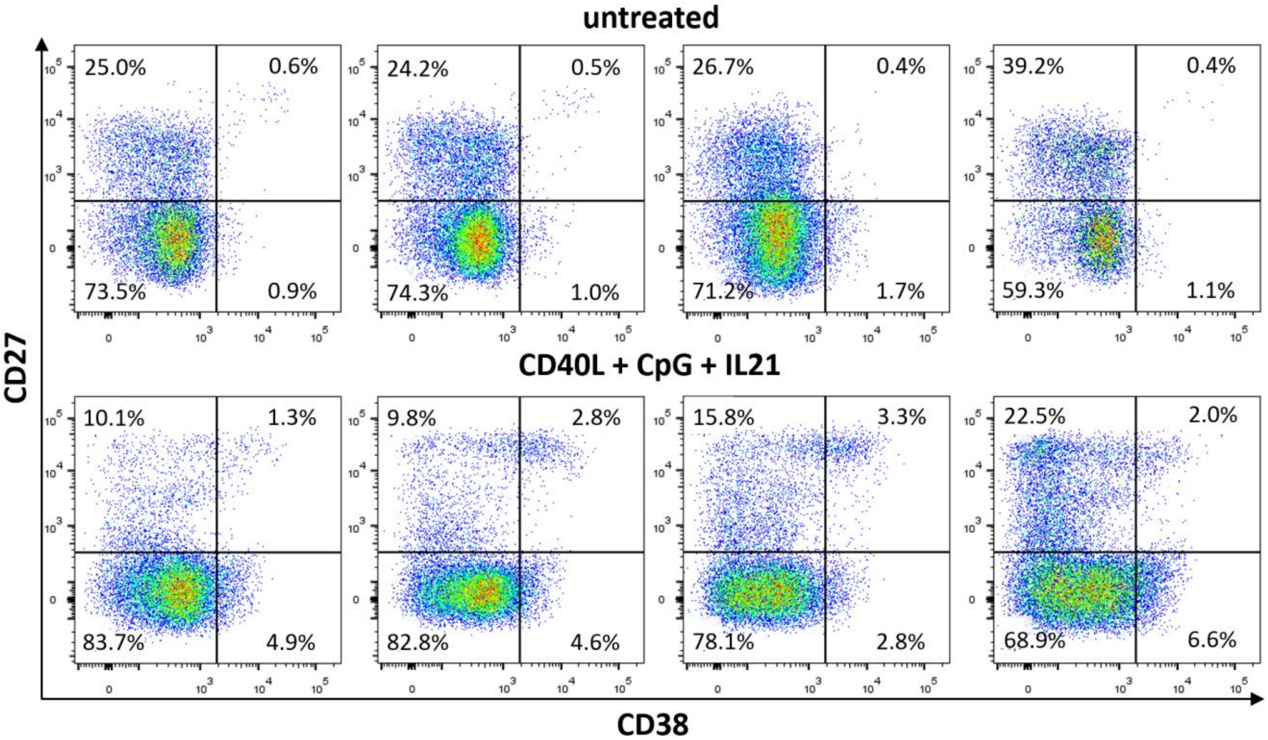

Figure S4. Distribution of CD27$^{–}$CD38^{int} (naïve B cells), CD27^{+}CD38$^{+/–}$ (memory B cells), and CD27^{hi}CD38^{hi} (plasmablasts) B cell subsets after 5 days of stimulation with CD40L + CpG + IL21 or without any activation.
